# The role of the gut microbiome in the association between habitual anthocyanin intake and visceral abdominal fat in population-level analysis

**DOI:** 10.1093/ajcn/nqz299

**Published:** 2019-12-11

**Authors:** Amy Jennings, Manja Koch, Majken K Jensen, Corinna Bang, Jan Kassubek, Hans-Peter Müller, Ute Nöthlings, Andre Franke, Wolfgang Lieb, Aedín Cassidy

**Affiliations:** 1 Department of Nutrition and Preventive Medicine, Norwich Medical School, University of East Anglia, Norwich, United Kingdom; 2 Department of Nutrition, Harvard TH Chan School of Public Health, Boston, MA, USA; 3 Institute of Epidemiology, Kiel University, Kiel, Germany; 4 Department of Medicine, Channing Division of Network Medicine, Harvard Medical School, Brigham and Women's Hospital, Boston, MA, USA; 5 Institute of Clinical Molecular Biology, Kiel University, Kiel, Germany; 6 Department of Neurology, University of Ulm, Ulm, Germany; 7 Department of Nutrition and Food Sciences, University of Bonn, Bonn, Germany; 8 Biobank PopGen, University Hospital Schleswig-Holstein, Kiel, Germany; 9 Institute for Global Food Security, Queen's University Belfast, Belfast, United Kingdom

**Keywords:** flavonoids, anthocyanins, gut microbiota, microbial diversity, abdominal adipose tissue, visceral fat, subcutaneous fat

## Abstract

**Background:**

Flavonoid intake modifies the composition of the gut microbiome, which contributes to the metabolism of flavonoids. Few studies have examined the contribution of the gut microbiome to the health benefits associated with flavonoid intake.

**Objectives:**

We aimed to examine associations between habitual intakes of flavonoid subclasses and MRI-determined visceral (VAT) and subcutaneous (SAT) adipose tissue. Uniquely, we also identified associations between the aforementioned measurements and gut microbiome composition sequenced from 16S ribosomal RNA genes.

**Methods:**

We undertook cross-sectional analyses of 618 men and women (*n* = 368 male), aged 25–83 y, from the PopGen cohort.

**Results:**

Higher intake of anthocyanins was associated with lower amounts of VAT [tertile (T)3-T1:  −0.49 dm^3^; β: −8.9%; 95% CI: −16.2%, −1.1%; *P *= 0.03] and VAT:SAT ratio (T3-T1: −0.04; β: −7.1%; 95% CI: −13.5%, −0.3%; *P *= 0.03). Higher intakes of anthocyanin-rich foods were also inversely associated with VAT [quantile (Q)4-Q1: −0.39 dm^3^; β: −9.9%; 95% CI: −17.4%, −1.6%; *P *= 0.02] and VAT:SAT ratio (Q4-Q1: −0.04; β: −6.5%; 95% CI: −13.3%, −0.9%; *P *= 0.03). Participants with the highest intakes of anthocyanin-rich foods also had higher microbial diversity (Q4-Q1: β: 0.18; 95% CI: 0.06, 0.31; *P* < 0.01), higher abundances of Clostridiales (Q4-Q1: β: 449; 95% CI: 96.3, 801; *P *= 0.04) and Ruminococcaceae (Q4-Q1: β: 313; 95% CI: 33.6, 591; *P *= 0.04), and lower abundance of Clostridium XIVa (Q4-Q1: β: −41.1; 95% CI: −72.4, −9.8; *P *= 0.04). Participants with the highest microbial diversity, abundances of Clostridiales and Ruminococcaceae, and lower abundance of Clostridium XIVa had lower amounts of VAT. Up to 18.5% of the association between intake of anthocyanin-rich foods and VAT could be explained by the gut microbiome.

**Conclusions:**

These novel data suggest that higher microbial diversity and abundance of specific taxa in the Clostridiales order may contribute to the association between higher intake of anthocyanins and lower abdominal adipose tissue.

## Introduction

The composition of the gut microbiome is a complex trait, shaped in part by environmental factors, with >20% of interindividual microbiome variability being related to diet and anthropometric factors (1, 2), which dominates over the role of genetics (1, 3). There is growing evidence showing that the gut microbiome plays an important role in the bioactivity of plant-derived dietary components called flavonoids (4). Flavonoids are a diverse range of bioactive compounds present in many commonly consumed fruits, vegetables, grains, herbs, and beverages (including tea, wine, and fruit juices) (5).

Flavonoids share a common basic chemical structure but are subdivided into subclasses based on the structural features of the heterocyclic ring, hydroxylation patterns, and the arrangement of functional groups within the flavonoid structure (5). These 6 subclasses [flavonols, flavones, flavanones, flavan-3-ols, anthocyanins, and flavonoid polymers (procyanidins and other polymers)] vary in functionality and biological efficacy. The majority of these flavonoid subclasses undergo extensive metabolism by colonic bacteria after ingestion (4). In addition, the gut microbiome plays a role in the bioavailability and metabolism of flavonoids, producing metabolites with increased bioactivity (4). In clinical trials, daily consumption of red wine polyphenols for 4 wk significantly increased the numbers of *Enterococcus*, *Prevotella*, *Bacteroides*, *Bifidobacterium*, *Eggerthella lenta*, and *Blautia coccoides–Eubacterium rectale* (6), whereas consumption of a high-cocoa flavanol drink for 4 wk significantly increased the abundance of *Bifidobacterium* and *Lactobacillus* species (quantified using 16S ribosomal RNA sequences) and decreased the abundance of *Clostridium histolyticum* (7). Both studies reported parallel beneficial changes in plasma lipid and C-reactive protein (CRP) concentrations, with changes in CRP linked to changes in abundance of *Bifidobacterium* and *Lactobacillus* species (6, 7). Animal studies have shown flavonoid-containing foods protect from diet-induced obesity by modulating the gut microbiome, most notably by increasing the relative abundance of *Akkermansia muciniphila* (8, 9).

It has been reported that higher intakes of flavonols, flavan-3-ols, anthocyanins, and the polymer subclasses are associated with less weight gain over the adult life course (10, 11) and that higher habitual intakes of both anthocyanins and flavonols were associated with lower fat mass and fat mass distribution (12). However, data on the associations between flavonoid intake and defined adipose tissues, including visceral (VAT) and subcutaneous (SAT) abdominal adipose tissue, are limited. In mice with diet-induced obesity, citrus flavonoid supplementation reduced VAT by 38–53% and SAT by 23–43% (13).

In the current study we aimed to further explore the associations between dietary flavonoid intake, the gut microbiome, and defined MRI measures of adiposity in humans. A priori we hypothesized significant associations between the anthocyanin subclass, abdominal fat, and the microbiome. Anthocyanins are extensively metabolized in the gut and epidemiological evidence indicates strong associations between higher anthocyanin intakes and improved measures of body fat (11, 12). Therefore, we first examined the associations between intakes of different flavonoid subclasses [flavanones, anthocyanins, flavan-3-ols, flavonols, flavones, polymeric flavonoids (and proanthocyanidins separately)] and *1*) MRI-determined volumes of VAT and SAT and *2*) gut microbiome diversity and abundances of taxa. Secondly, we investigated if the gut microbiome influenced the proposed associations between intake of flavonoid subclasses and abdominal adipose tissue.

## Methods

### Study population

Participants were from the PopGen control cohort, a community-based sample of 1316 men and women recruited from the general population in Kiel, northern Germany, between 2005 and 2007 (14). The PopGen biobank randomly identified 23,000 local residents through population registries in the city of Kiel and invited them to participate in the study. A total of 4267 participants agreed to take part and, among them, 747 subjects agreed to participate in the follow-up study. In addition, the PopGen biobank recruited 569 blood donors at the University Hospital Schleswig-Holstein in Kiel, and these 2 groups constituted the final PopGen control cohort (1316 individuals). The first follow-up examination of these participants took place between 2010 and 2012 and 929 participants agreed to be re-examined. This follow-up examination included biochemical, phenotypic, and dietary assessments and the collection of biological samples, including blood and stool samples. A total of 656 participants agreed to participate in a whole-body MRI examination. Complete data on the MRI variables were available for 626 participants, of which we excluded those who were missing dietary (*n* = 1) or microbiome (*n* = 7) data (**Supplemental Figure 1**). All participants were unaware of the specific hypotheses being tested and were not selected for particular diseases or traits. The study was approved by the Christian-Albrechts University of Kiel ethical review board and all subjects provided informed written consent.

### Adipose tissue assessment

Whole-body MRI was performed on a Magnetom Avanto 1.5-Tesla scanner (Siemens Medical Solutions) with participants in a supine position with arms extended above the head and required to hold their breath to minimize breathing motion artefacts. Transversal images were obtained by a T1-weighted gradient echo sequence (repetition time 157 ms, time to echo 4 ms, flip angle 70°, voxel size 3.9 × 2.0 × 8.0 mm^3^). Continuous cross-sectional images with 8-mm slice thickness and 2-mm interslice gaps were obtained in the thoracic and abdominal regions.

Data preprocessing and analysis were conducted by the semiautomatic software package ATLAS (Automatic Tissue Labeling Analysis Software; University of Ulm) (15). Segmentation of VAT and SAT voxels was performed by using the Adapted Rendering for Tissue Intensity Segmentation algorithm as previously described (15, 16); both VAT and SAT voxels were measured from the top of the liver to the femoral heads. The number of voxels was multiplied by the voxel size to obtain the volume (dm^3^) of VAT and SAT. During postprocessing, liver fat, fat in the intestinal loops, and minor MRI artefacts caused mainly by hip implants and stents were excluded from segmented VAT. If MRI artefacts were present that could not be corrected for, such as extensive breathing motion, participants were excluded from further analysis (*n* = 30). The VAT:SAT ratio, a metric of relative body fat composition that quantifies the propensity to store fat viscerally relative to subcutaneous fat, was calculated as the volume of VAT over SAT (17).

### Dietary assessment

Dietary intake over the previous year was calculated using a self-administered 112-item FFQ, originally designed and validated for use in the German European Prospective Investigation into Cancer and Nutrition (EPIC) study (18). Flavonoid content values were assigned to each of the foods listed in the FFQ using data from the USDA as the primary data source (19, 20). For foods assessed in the FFQ without values in the USDA database, we searched Phenol-Explorer (www.phenol-explorer.eu) to ensure all available high-quality data on flavonoid values were included. Intakes were derived for the main subclasses of flavonoids habitually consumed: flavanones (eriodictyol, hesperetin, and naringenin), anthocyanins (cyanidin, delphinidin, malvidin, pelargonidin, petunidin, and peonidin), flavan-3-ols (catechins and epicatechins), flavonols (quercetin, kaempferol, myricetin, and isohamnetin), flavones (luteolin and apigenin), polymers [including proanthocyanidins (excluding monomers), theaflavins, and thearubigins], and proanthocyanidins separately (dimers, trimers, 4–6-mers, 7–10-mers, polymers, and monomers). Total flavonoid intakes were calculated by summing the 6 component subclasses (flavanones, anthocyanins, flavan-3-ols, flavonols, flavones, and polymers). Intakes of energy and other nutrients were determined using values from the German Food Code and Nutrient Database (version II.3) (21).

### Gut microbiome composition analysis

Fecal bacterial DNA was extracted using the QIAamp DNA Stool Mini kit (Qiagen) on a QIAcube system. After extraction, the V1–V2 region of the 16S ribosomal RNA gene was sequenced on the MiSeq (Illumina Inc.) platform, using the 27F–338R primer pair and dual multiple identifer indexing (8 nucleotides each on the forward and reverse primers) with the MiSeq Reagent Kit version 3 as previously described (22). After sequencing, MiSeq fastq files were derived from base calls for reads 1 and 2, as well as indexes 1 and 2, using the Bcl2fastq module in CASAVA 1.8.2 with no mismatches in either index sequence allowed. Forward and reverse reads were merged with FLASH software (version 1.2) (23) and high-quality data were derived (sequences with <5% nucleotides with a quality score >30 performed with the fastx toolkit). After removing chimeras in sequences using UCHIME (version 6.0), 10,000 reads for each sample were randomly selected (24). Sequences were clustered at each taxonomic level using the Ribosomal Database Project classifier with the latest reference database (version 14) (25). Classifications with low confidence at the genus level (<0.8) were organized in an arbitrary taxon of “unclassified family.” Genus-level operational taxonomic units (97% similarity) were created using the UPARSE routine (26).

### Covariate assessment

Data on participant characteristics, including sex, age, smoking status, education, use of dietary supplements, and physical activity, were collected by self-administered questionnaire.

For physical activity participants were asked to report the time spent walking, cycling, engaging in sports and gardening (average of summer and winter seasons), household work, and do-it-yourself activities per week over the past year and the number of flights of stairs climbed per day. The duration of each physical activity was multiplied by the corresponding metabolic equivalent intensity level and the products were summed for all activities (27). Weight and height were measured with subjects dressed in light clothing without shoes; 2 kg were subtracted to account for clothing. We calculated the ratio of energy intake to estimated energy requirement (EER) as follows: 100×[energy intake (kcal)/EER]. EER was determined using Institute of Medicine equations, by BMI (in kg/m^2^), age, and sex (28).

### Statistical analysis

We compared dietary and lifestyle characteristics between men and women using independent *t* tests (for continuous traits) or chi-squared tests (for categorical data). Participants were ranked into tertiles (Ts) of estimated intake for the flavonoid subclasses and associations with MRI-determined volumes of VAT, SAT, and VAT:SAT ratio were assessed using ANCOVA. If a subclass was found to be significantly associated with any of the outcomes (VAT, SAT, or VAT:SAT ratio), we also examined the associations with foods known to contribute considerably to intake of the respective subclass. We calculated the contribution of each food group to the intake of the relevant subclass. These foods were combined to calculate total intake of foods rich in a particular subclass and participants were categorized into quantiles (Qs) of intake (quantiles rather than tertiles of food intake were used to ensure equal numbers of participants in each of the categories). We checked for effect modification by including interaction terms for sex/menopausal status (male, premenopausal females, postmenopausal females) and flavonoid intake in the models.

We then examined the association between intake of the flavonoid subclasses and flavonoid-rich foods and both taxa abundances and microbial diversity, using the Shannon index and the Bray–Curtis dissimilarity measure, using ANCOVA. In these analyses, we considered the first 3 principal components from the principal coordinate analysis. We focused on those flavonoid subclasses that had provided evidence for association with MRI-derived adiposity measures in the first analysis.

To reduce random error in low-abundance taxa we focused our analysis on the core measurable microbiota, determined as highly reproducible taxa (*r*^2^ > 0.97), which in this data set were those with >40 reads per replicate in 10,000 reads (64 taxa across 5 levels; phylum, class, order, family, and genus) (3). We further filtered abundances by excluding taxa where >60% of the counts were 0 (*n* = 2 taxa). Because some higher taxonomic levels consisted of only a single genus we removed summarized taxa with a correlation ≥0.9 to prevent duplication. If we observed significant associations of flavonoid subclasses with diversity measures or taxa, we then assessed the relation between these microbial factors and MRI-determined VAT, SAT, and their ratio using ANCOVA.

All models were adjusted for sex; age (y); smoking (never, former, current); physical activity (metabolic equivalents per week); height (cm); use of vitamin or mineral supplements (yes or no); education level (none or primary/middle school, secondary school or college/further education); and daily intakes (in Ts) of energy (kcal), polyunsaturated, monounsaturated, and saturated fat (g/d), fiber (g/d), alcohol (g/d), and carbonated drinks (frequency per d). We also included the ratio of energy intake to EER as a measure of energy misreporting. For the models assessing the association between flavonoid intakes and microbial factors we also included BMI as a covariate. Ln-transformed values were used for MRI-derived fat values which are presented as geometric means (95% CIs), or, where the difference between extreme quantiles is reported, as the percentage change in the dependent variable per unit change in the independent variable {100×[exp(β) – 1]}.

We used structural equation modeling to quantify the amount of variation in the association between flavonoid intake and abdominal fat outcomes that is explained by the microbiome. We combined any gut microbiome diversity variables or abundances of taxa associated with both flavonoid intake and MRI-derived fat values using principal component analysis in order to assess the combined function of the microbiome. We presented the results as a ratio of the indirect association [the association between flavonoid intake (exposure) and MRI-determined fat measures (outcome) mediated by the microbiome] to the total association [association between flavonoid intake and the microbiome on the outcome variable (MRI-derived fat values)]. This represented the proportion of the variance explained by the mediating variable.

Two-sided *P* values < 0.05 were considered statistically significant for all analyses with the exception of the microbial taxa abundances, where a multiple testing correction was applied using the Benjamini–Hochberg method for false discovery rate where a *Q* value < 0.05 was considered statistically significant. Statistical analyses were performed with Stata statistical software version 15 (StataCorp).

## Results

The demographic characteristics and dietary intakes of the 618 participants, aged 25–83 y, are shown in **Table 1**. Mean ± SD total estimated flavonoid intake was 765 ± 673 mg/d and anthocyanin intake 41.3 ± 40.5 mg/d.

**TABLE 1:** Characteristics and dietary intakes of PopGen participants by sex^1^

Variable	Male (*n* = 368)	Female (*n* = 250)	*P*
Age, y	61.5 ± 11.1	60.8 ± 12.7	0.47
Physical activity, MET/wk	98.1 ± 65.5	116 ± 61.1	<0.01
Current smoker (yes)	39 (54.2)	33 (45.8)	<0.01
Vitamin supplement use (yes)	109 (44.3)	137 (55.7)	<0.01
SAT, dm^3^	8.8 ± 3.7	11.4 ± 5.4	<0.01
VAT, dm^3^	5.0 ± 2.1	2.9 ± 1.5	<0.01
SAT:VAT ratio	0.84 ± 0.27	0.38 ± 0.13	<0.01
BMI, kg/m^2^	27.4 ± 3.8	26.8 ± 5.2	0.08
Height, cm	178 ± 7.2	163 ± 7.3	<0.01
Total flavonoids, mg/d	751 ± 688	786 ± 652	0.52
Flavanones, mg/d	24.0 ± 29.9	19.9 ± 19.9	0.06
Anthocyanins, mg/d	39.0 ± 36.1	44.5 ± 46.0	0.10
Flavan-3-ols, mg/d	221 ± 329	230 ± 309	0.72
Flavonols, mg/d	32.8 ± 25.9	30.8 ± 23.1	0.32
Flavones, mg/d	4.8 ± 3.3	4.3 ± 3.4	0.09
Polymers, mg/d	430 ± 355	456 ± 331	0.34
Proanthocyanidins, mg/d	543 ± 446	586 ± 445	0.24
Energy, kcal/d	2519 ± 690	1967 ± 507	<0.01
PUFAs, g/d	19.6 ± 6.7	15.7 ± 4.9	<0.01
SFAs, g/d	44.7 ± 14.2	35.0 ± 10.8	<0.01
MUFAs, g/d	39.9 ± 11.7	30.4 ± 8.9	<0.01
Fiber, g/d	23.3 ± 7.5	21.5 ± 6.1	<0.01
Alcohol, g/d	20.0 ± 22.6	9.7 ± 11.8	<0.01
Carbonated drinks, freq/d	0.37 ± 0.92	0.33 ± 0.80	0.58
Ratio of energy intake to EER, %	93.0 ± 27.1	98.2 ± 27.9	0.02

1 *n* = 618. Values are mean ± SD or *n* (%). *P* values are for the differences between men and women calculated from independent *t* tests for continuous data and chi-squared tests for categorical data. EER, estimated energy requirement; MET, metabolic equivalent; SAT, subcutaneous abdominal adipose tissue; VAT, visceral abdominal adipose tissue.

### Associations between flavonoid subclasses, flavonoid-rich foods, and abdominal fat

A higher habitual intake of anthocyanins was associated with lower amounts of VAT (T3-T1: −0.49 dm^3^; β: −8.9%; 95% CI: −16.2%, −1.1%; *P = *0.03) and a lower ratio of VAT to SAT (T3-T1: −0.04; β: −7.1%; 95% CI: −13.5%, −0.3%; *P *= 0.04) (**Table 2**). There was a significant interaction between sex/menopausal status and anthocyanin intake in the VAT model due to a greater association in men (β: −0.08, *P *< 0.01) than in pre- (β: −0.05, *P *= 0.37) and postmenopausal women (β: 0.02, *P *= 0.53).

**TABLE 2:** MRI-determined volumes of VAT and SAT and their ratio by quantiles of flavonoid subclass intake in PopGen participants^1^

	Quantile 1	Quantile 2	Quantile 3	*P*
SAT, dm^3^				
Total flavonoids	6.8 (6.4, 7.3)	6.5 (6.2, 6.9)	6.5 (6.1, 6.9)	0.56
Flavanones	6.7 (6.4, 7.1)	6.4 (6.1, 6.8)	6.7 (6.3, 7.1)	0.23
Anthocyanins	6.9 (6.5, 7.3)	6.4 (6.1, 6.8)	6.5 (6.1, 6.9)	0.64
Flavan-3-ols	6.9 (6.5, 7.4)	6.4 (6.1, 6.8)	6.5 (6.1, 6.9)	0.23
Flavonols	6.9 (6.5, 7.3)	6.4 (6.1, 6.8)	6.5 (6.1, 6.9)	0.64
Flavones	6.6 (6.2, 7.0)	6.8 (6.4, 7.2)	6.5 (6.1, 6.9)	0.66
Polymers	6.8 (6.4, 7.2)	6.5 (6.1, 6.9)	6.5 (6.1, 6.9)	0.89
Proanthocyanidins	6.9 (6.5, 7.3)	6.5 (6.1, 6.9)	6.5 (6.1, 6.9)	0.56
VAT, dm^3^				
Total flavonoids	3.9 (3.6, 4.1)	3.6 (3.4, 3.9)	3.7 (3.4, 3.9)	0.61
Flavanones	3.8 (3.5, 4.0)	3.7 (3.5, 3.9)	3.7 (3.4, 3.9)	0.67
Anthocyanins	4.0 (3.7, 4.3)	3.6 (3.4, 3.9)	3.5 (3.3, 3.8)	0.03
Flavan-3-ols	3.9 (3.6, 4.2)	3.7 (3.5, 3.9)	3.6 (3.3, 3.8)	0.10
Flavonols	3.8 (3.5, 4.1)	3.6 (3.4, 3.8)	3.8 (3.5, 4.1)	0.34
Flavones	3.8 (3.5, 4.0)	3.8 (3.6, 4.1)	3.5 (3.3, 3.8)	0.43
Polymers	3.9 (3.6, 4.1)	3.7 (3.4, 3.9)	3.6 (3.4, 3.9)	0.81
Proanthocyanidins	4.0 (3.7, 4.2)	3.6 (3.4, 3.9)	3.6 (3.3, 3.9)	0.19
VAT:SAT, dm^3^				
Total flavonoids	0.57 (0.54, 0.61)	0.56 (0.52, 0.59)	0.57 (0.54, 0.61)	0.99
Flavanones	0.56 (0.53, 0.60)	0.58 (0.55, 0.62)	0.55 (0.52, 0.59)	0.45
Anthocyanins	0.58 (0.55, 0.62)	0.57 (0.54, 0.61)	0.54 (0.51, 0.58)	0.04
Flavan-3-ols	0.57 (0.53, 0.60)	0.58 (0.55, 0.61)	0.55 (0.52, 0.59)	0.49
Flavonols	0.55 (0.52, 0.59)	0.56 (0.53, 0.59)	0.59 (0.55, 0.63)	0.11
Flavones	0.58 (0.54, 0.61)	0.57 (0.54, 0.61)	0.55 (0.52, 0.59)	0.17
Polymers	0.57 (0.54, 0.61)	0.57 (0.53, 0.60)	0.56 (0.53, 0.60)	0.67
Proanthocyanidins	0.58 (0.55, 0.61)	0.56 (0.53, 0.59)	0.56 (0.52, 0.60)	0.35

1 *n* = 618. Values are geometric means (95% CIs). Models were adjusted for sex; age (y); smoking (never, former, current); physical activity (metabolic equivalents per week); height (cm); use of vitamin or mineral supplements (yes or no); education level (none or primary/middle school, secondary school or college/further education); daily intakes (in tertiles) of energy (kcal), polyunsaturated, monounsaturated, and saturated fat (g/d), fiber (g/d), alcohol (g/d), and carbonated drinks (frequency/d); and the ratio of energy intake to estimated energy requirements. *P* value calculated using ANCOVA. SAT, subcutaneous abdominal adipose tissue; VAT, visceral abdominal adipose tissue.

A higher intake of foods rich in anthocyanins [combined intake of strawberries, other berries (red currants, blackberries, and blueberries), and red wine] was also associated with lower amounts of VAT (Q4-Q1: −0.39 dm^3^; β: −9.9%; 95% CI: −17.4%, −1.6%; *P *= 0.02) and VAT:SAT ratio (Q4-Q1: −0.04; β: −6.5%; 95% CI: −13.3%, −0.9%; *P *= 0.03) (**Table 3**). No significant interactions between sex/menopausal status and foods rich in anthocyanins were observed. Mean frequency of strawberry consumption was 0.5 portions/d, other berries 0.2 portions/d, and red wine 0.1 portions/d (data not shown). These observations were made after adjustment for a number of lifestyle and dietary covariates including smoking status, physical activity, and intakes of energy, individual fatty acids, fiber, alcohol, and carbonated drinks. We observed no significant associations between other flavonoid subclasses and VAT, SAT, or their ratio.

**TABLE 3:** MRI-determined volumes of VAT and SAT and their ratio by quantiles of the intake of anthocyanin-rich foods in PopGen participants^1^

	Quantile 1	Quantile 2	Quantile 3	Quantile 4	*P*
SAT, dm^3^	6.5 (6.2, 6.9)	5.9 (5.6, 6.2)	6.0 (5.7, 6.3)	6.3 (5.9, 6.6)	0.60
VAT, dm^3^	3.9 (3.7, 4.2)	3.5 (3.3, 3.7)	3.3 (3.2, 3.5)	3.5 (3.3, 3.7)	0.02
VAT:SAT, dm^3^	0.60 (0.57, 0.64)	0.60 (0.57, 0.63)	0.56 (0.53, 0.58)	0.56 (0.54, 0.59)	0.03

1 *n* = 618. Values are geometric means (95% CIs). Models adjusted for sex; age (y); smoking (never, former, current); physical activity (metabolic equivalents per week); height (cm); use of vitamin or mineral supplements (yes or no); education level (none or primary/middle school, secondary school or college/further education); daily intakes (in tertiles) of energy (kcal), polyunsaturated, monounsaturated, and saturated fat (g/d), fiber (g/d), alcohol (g/d), and carbonated drinks (frequency/d); and the ratio of energy intake to estimated energy requirements. *P* value calculated using ANCOVA. Foods rich in anthocyanins include red wine, strawberries, and other berries (red currants, blackberries, blueberries). SAT, subcutaneous abdominal adipose tissue; VAT, visceral abdominal adipose tissue.

### Associations between flavonoid subclasses, flavonoid-rich foods, and microbial diversity

Higher intakes of anthocyanins (T3-T1: 0.12; 95% CI: 0.01, 0.23; *P *= 0.04) and foods rich in anthocyanins (Q4-Q1: 0.18; 95% CI: 0.06, 0.31; *P* < 0.01) were associated with greater microbial diversity, as measured by the Shannon index (**Table 4**). The second and third principal components of the Bray–Curtis measure of microbial dissimilarity were associated with intake of anthocyanin-rich foods (*P *= 0.04 and 0.03, respectively) but not anthocyanin intake (*P *= 0.89 and 0.65, respectively).

**TABLE 4:** Measures of α-microbial diversity (Shannon index) and β-microbial diversity (Bray–Curtis index) by quantiles of the intake of anthocyanins and foods rich in anthocyanins in PopGen participants^1^

	Quantile 1	Quantile 2	Quantile 3	Quantile 4	*P*
Anthocyanins					
Shannon index	4.2 (4.1, 4.3)	4.3 (4.2, 4.3)	4.3 (4.2, 4.4)		0.04
Bray–Curtis (PCoA-1)	0.01 (−0.08, 0.11)	0.02 (−0.06, 0.11)	0.04 (−0.05, 0.14)	—	0.64
Bray–Curtis (PCoA-2)	−0.01 (−0.10, 0.08)	−0.05 (−0.14, 0.03)	−0.02 (−0.11, 0.07)	—	0.89
Bray–Curtis (PCoA-3)	0.01 (−0.08, 0.10)	0.09 (0.01, 0.18)	−0.02 (−0.11, 0.07)	—	0.65
Anthocyanin-rich foods					
Shannon index	4.2 (4.1, 4.2)	4.2 (4.1, 4.3)	4.3 (4.2, 4.4)	4.4 (4.3, 4.4)	<0.01
Bray–Curtis (PCoA-1)	−0.05 (−0.16, 0.06)	0.07 (−0.03, 0.16)	0.05 (−0.05, 0.15)	0.03 (−0.07, 0.13)	0.43
Bray–Curtis (PCoA-2)	0.04 (−0.07, 0.15)	0.03 (−0.07, 0.12)	−0.09 (−0.19, 0.01)	−0.09 (−0.19, 0.01)	0.04
Bray–Curtis (PCoA-3)	−0.11 (−0.21, 0.00)	0.04 (−0.06, 0.13)	0.12 (0.02, 0.22)	0.05 (−0.05, 0.15)	0.03

1 *n* = 618. Values are means (95% CIs). Models adjusted for sex; age (y); smoking (never, former, current); physical activity (metabolic equivalents per week); BMI (kg/m^2^); height (cm); use of vitamin or mineral supplements (yes or no); education level (none or primary/middle school, secondary school or college/further education); daily intakes (in tertiles) of energy (kcal), polyunsaturated, monounsaturated, and saturated fat (g/d), fiber (g/d), alcohol (g/d), and carbonated drinks (frequency/d); and the ratio of energy intake to estimated energy requirements. *P* value calculated using ANCOVA. Foods rich in anthocyanins include red wine, strawberries, and other berries (red currants, blackberries, blueberries). PCoA, principal coordinate analysis.

### Associations between flavonoid subclasses, flavonoid-rich foods, and taxa abundance

**Supplemental Figure 2** shows the relative abundances of the taxa (by genus and family) that make up the core measurable data set. Examining the 4 major bacterial phyla we identified a positive association between a higher intake of anthocyanin-rich foods and abundance of Firmicutes (Q4-Q1: 502; 95% CI: 110, 894; *Q* = 0.03) (**Table 5**). Examining the bacterial taxa within this phyla (14 taxa across 3 levels; 2 order, 4 family, and 8 genus) revealed a relation between higher intake of anthocyanin-rich foods and higher abundance of Clostridiales (Q4-Q1: 449; 95% CI: 96.3, 801; *P *= 0.04). Of the families in the Clostridiales order, higher intake of anthocyanin-rich foods was associated with Ruminococcaceae (Q4-Q1: 313; 95% CI: 33.6, 591; *P *= 0.04). At a higher taxonomic level, higher intake of anthocyanin-rich foods was associated with lower abundances of the genera Clostridium XIVa (Q4-Q1: −41.1; 95% CI: −72.4, −9.8; *P *= 0.01, *Q* = 0.04), unclassified Ruminococcaceae (Q4-Q1: 178; 95% CI: 31.3, 387; *P *= 0.03, *Q* = 0.07), and *Roseburia* (Q4-Q1: 52.3; 95% CI: 10.6, 94.0; *P *= 0.02, *Q* = 0.06), although the latter 2 associations did not meet the significance threshold after adjustment for multiple testing. Further adjustment for α-diversity did not materially alter the results for Clostridiales (Q4-Q1: 401; 95% CI: 50.9, 750; *P* = 0.01), Ruminococcaceae (Q4-Q1: 269; 95% CI: −7.2, 544; *P* = 0.02), and Clostridium XIVa (Q4-Q1: −37.4; 95% CI: −68.6, −6.2; *P* = 0.02) (data not shown). We observed no associations between anthocyanin intake and abundance of taxa (data not shown).

**TABLE 5:** Abundances of 4 major bacterial phyla and of taxa within the Firmicutes phylum by quantiles of intake of foods rich in anthocyanins in PopGen participants^1^

	Quantile 1	Quantile 2	Quantile 3	Quantile 4	*P*	*Q*
Actinobacteria	134 (99.5, 168)	189 (159, 219)	142 (111, 174)	156 (124, 188)	0.88	0.99
Bacteroidetes	3749 (3479, 4020)	3434 (3196, 3672)	3354 (3105, 3603)	3418 (3165, 3672)	0.11	0.22
Firmicutes	5146 (4872, 5420)	5428 (5188, 5669)	5769 (5517, 6021)	5648 (5392, 5905)	0.01	0.03
Proteobacteria	945 (757, 1134)	927 (761, 1093)	703 (529, 876)	755 (578, 931)	0.07	0.22
Lactobacillales	105 (69.0, 141)	136 (105, 168)	145 (113, 178)	141 (107, 174)	0.18	0.26
Clostridiales	4145 (3899, 4392)	4329 (4112, 4545)	4672 (4445, 4899)	4594 (4363, 4825)	0.00	0.04
*Blautia*	181 (140, 222)	202 (166, 238)	193 (156, 231)	193 (154, 231)	0.82	0.82
Clostridium XIVa	72.5 (50.6, 94.4)	47.2 (28.0, 66.4)	33.9 (13.8, 54.0)	31.5 (10.9, 52.0)	0.01	0.04
*Roseburia*	95.5 (66.3, 125)	141 (115, 166)	155 (129, 182)	148 (120, 175)	0.02	0.06
Unclassified Lachnospiraceae	1083 (985, 1180)	1073 (988, 1158)	1142 (1052, 1231)	1158 (1067, 1249)	0.18	0.26
Ruminococcaceae	2434 (2239, 2629)	2525 (2354, 2696)	2789 (2610, 2969)	2746 (2564, 2929)	0.01	0.04
*Fecalibacterium*	550 (448, 651)	619 (530, 708)	614 (521, 707)	588 (493, 682)	0.70	0.75
*Oscillibacter*	338 (285, 392)	321 (274, 368)	357 (308, 406)	370 (320, 420)	0.27	0.34
*Ruminococcus*	168 (118, 218)	185 (142, 229)	206 (160, 252)	216 (169, 262)	0.16	0.26
Unclassified Ruminococcaceae	1267 (1121, 1414)	1295 (1167, 1424)	1513 (1378, 1647)	1445 (1308, 1582)	0.03	0.07
Unclassified Erysipelotrichaceae	163 (127, 200)	165 (133, 197)	152 (119, 186)	180 (146, 215)	0.60	0.70
Acidaminococcaceae	221 (169, 272)	144 (98.1, 189)	128 (80.8, 176)	133 (84.0, 181)	0.03	0.07
*Dialister*	321 (188, 453)	471 (354, 587)	541 (419, 663)	476 (352, 601)	0.11	0.21

1 *n* = 618. Values are means (95% CIs). Models adjusted for sex; age (y); smoking (never, former, current); physical activity (metabolic equivalents per week); BMI (kg/m^2^); height (cm); use of vitamin or mineral supplements (yes or no); education level (none or primary/middle school, secondary school or college/further education); daily intakes (in tertiles) of energy (kcal), polyunsaturated, monounsaturated, and saturated fat (g/d), fiber (g/d), alcohol (g/d), and carbonated drinks (frequency/d); and the ratio of energy intake to estimated energy requirements. *P* value and *Q* value (FDR-adjusted *P* value) calculated using ANCOVA.

### Associations between microbial diversity and abdominal fat

Participants with the highest α-diversity (as measured by the Shannon index) had significantly lower VAT (T3-T1: −0.33 dm^3^; β: −7.2%; 95% CI: −13.3%, −0.6%; *P* = 0.03) (**Table 6**). There was also an association between β-diversity (measured using the Bray–Curtis index) and VAT (T3-T1: 0.46 dm^3^; β: 12.5%; 95% CI: 5.1%, 20.5%; *P* < 0.01). Higher abundances of Clostridiales (T3-T1: −0.33 dm^3^; β: −7.6%; 95% CI: −13.7%, −1.1%; *P *= 0.02) and Ruminococcaceae (T3-T1: −0.35 dm^3^; β: −7.6%; 95% CI: −13.7%, −1.1%; *P *= 0.02) were also associated with significantly lower VAT. Conversely, a higher abundance of Clostridium XIVa was associated with significantly higher VAT (T3-T1: 0.49 dm^3^; β: 13.6%; 95% CI: 6.1%, 21.6%; *P* < 0.01). There were no associations between microbial factors and the ratio of VAT to SAT. Adjustment for intake of anthocyanin-rich food did not markedly change the results (data not shown).

**TABLE 6:** MRI-determined volume of VAT and the ratio of VAT to SAT by quantiles of microbial diversity and abundance in PopGen participants^1^

	Quantile 1	Quantile 2	Quantile 3	*P*
VAT				
Shannon index	3.7 (3.4, 4.2)	3.6 (3.2, 4.1)	3.4 (3.1, 3.8)	0.03
Bray–Curtis (PCoA-2)	3.4 (3.0, 3.8)	3.6 (3.2, 4.0)	3.8 (3.4, 4.3)	<0.01
Bray–Curtis (PCoA-3)	3.5 (3.2, 4.0)	3.6 (3.2, 4.0)	3.7 (3.3, 4.1)	0.33
Clostridiales	3.8 (3.4, 4.2)	3.5 (3.1, 3.9)	3.5 (3.1, 3.9)	0.02
Clostridium XIVa	3.3 (3.0, 3.7)	3.7 (3.3, 4.1)	3.8 (3.4, 4.2)	<0.01
Ruminococcaceae	3.8 (3.4, 4.3)	3.5 (3.1, 3.9)	3.5 (3.1, 3.9)	0.02
VAT:SAT				
Shannon index	0.59 (0.53, 0.65)	0.57 (0.52, 0.63)	0.58 (0.52, 0.64)	0.58
Bray–Curtis (PCoA-2)	0.57 (0.52, 0.63)	0.56 (0.51, 0.62)	0.61 (0.55, 0.67)	0.07
Bray–Curtis (PCoA-3)	0.57 (0.52, 0.63)	0.58 (0.53, 0.64)	0.59 (0.53, 0.65)	0.40
Clostridiales	0.59 (0.54, 0.65)	0.58 (0.53, 0.64)	0.57 (0.52, 0.63)	0.27
Clostridium XIVa	0.57 (0.52, 0.63)	0.57 (0.52, 0.63)	0.60 (0.54, 0.66)	0.07
Ruminococcaceae	0.60 (0.55, 0.66)	0.57 (0.51, 0.62)	0.57 (0.52, 0.63)	0.17

1 *n* = 618. Values are geometric means (95% CIs). Models adjusted for sex; age (y); smoking (never, former, current); physical activity (metabolic equivalents per week); height (cm); use of vitamin or mineral supplements (yes or no); education level (none or primary/middle school, secondary school or college/further education); daily intakes (in tertiles) of energy (kcal), polyunsaturated, monounsaturated, and saturated fat (g/d), fiber (g/d), alcohol (g/d), and carbonated drinks (frequency/d); and the ratio of energy intake to estimated energy requirements. *P* value calculated using ANCOVA. PCoA, principal coordinate analysis; SAT, subcutaneous abdominal adipose tissue; VAT, visceral abdominal adipose tissue.

### Proportion of variance in the association between flavonoid intake and abdominal fat that is explained by the microbiome

**FIGURE 1:**
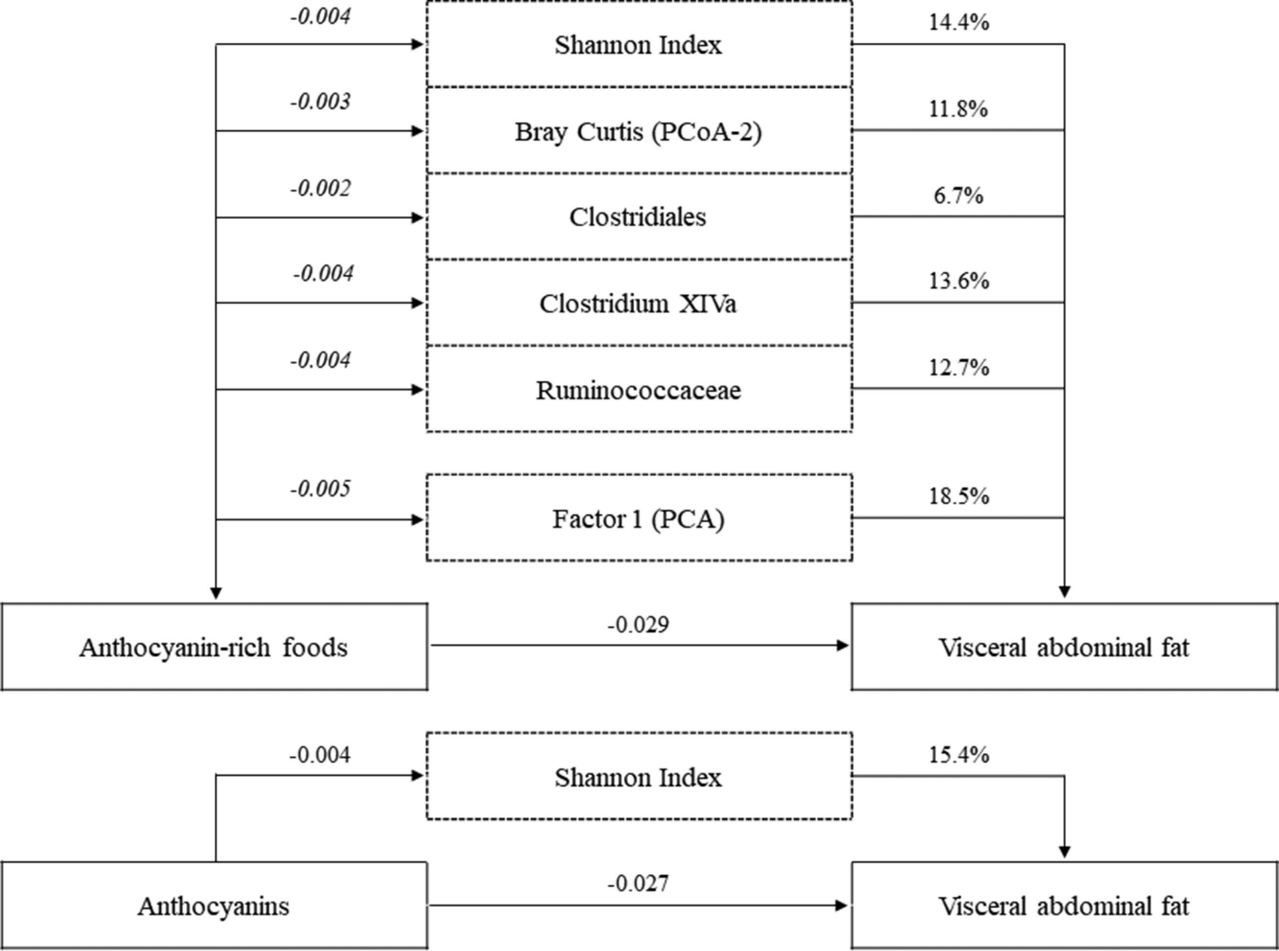
Path coefficients for associations between anthocyanin intake and volumes of VAT mediated by microbial factors in *n*= 618 PopGen participants. Values are the path coefficients, calculated using structural equation modeling, for the total and indirect associations between intakes of anthocyanins and anthocyanin-rich foods, microbial factors, and MRI-determined volumes of VAT. Values in bold represent the total association between intake and VAT controlling for microbial factors; values in italics represent the indirect association between intake and VAT explained by microbial factors; percentage values are the ratio of the indirect to the total association and represent the percentage of variation explained by the microbial factors. Factor 1 is the first principal component (58% of the variance) from analysis of all the microbial factors associated with both intake and VAT. Models adjusted for sex; age (y); smoking (never, former, current); physical activity (metabolic equivalents per week); height (cm); use of vitamin or mineral supplements (yes or no); education level (none or primary/middle school, secondary school or college/further education); daily intakes (in tertiles) of energy (kcal), polyunsaturated, monounsaturated, and saturated fat (g/d), fiber (g/d), alcohol (g/d), and carbonated drinks (frequency/d); and the ratio of energy intake to estimated energy requirements. PCA, Principal Component Analysis, PCoA, Principal Coordiante Analysis.

The proportion of the association between intake of anthocyanin-rich foods and VAT that could be explained by the gut microbiome was 14.4% for α-diversity, 11.8% for β-diversity, 6.7% for abundance of Clostridiales, 12.7% for abundance of Ruminococcaceae, and 13.6% for abundance of Clostridium XIVa (**Figure 1**). A linear combination of these gut microbiome variables (first principal component: 58% of the variance) explained 18.5% of the association between intake of anthocyanin-rich foods and VAT.

## Discussion

To our knowledge, this is the first study to examine associations and interrelations between flavonoid subclasses, objective MRI-determined visceral and subcutaneous fat volumes, and the gut microbiome. Our novel findings demonstrated that higher consumption of foods rich in anthocyanins (1.8 portion/d of strawberries, red currants, blackberries, blueberries, or red wine) was associated with lower VAT, greater microbial diversity as measured by the Shannon index, and higher abundance of Clostridiales and Ruminococcaceae and lower abundance of Clostridium XIVa. These microbiome variables were notable mediators, explaining 18.5% of the association between intake of anthocyanin-rich foods and VAT, with microbial diversity independently explaining 15% of the association. We were able to confirm that associations with microbial diversity were independent of individual taxa, suggesting that both abundance and richness of species are important factors in these interactions. This underlines the importance in future studies of recognizing that components of the microbial community are likely to act synergistically in metabolizing complex dietary components such as flavonoids.

The gut microbiome and flavonoid intake and metabolism are interrelated, with flavonoid intake altering the composition of the gut microbiome and the gut microbiome metabolizing flavonoids into compounds with enhanced bioactivity (4). Recent data show anthocyanin metabolites are major mediators of the metabolic benefits of anthocyanin-rich foods like blueberries in humans (29). These data are supported by mechanistic data showing that the bioactivity of anthocyanins is attributable to their microbially derived metabolites, which are present in the circulation significantly longer and at much higher concentrations than the parent anthocyanins, and exert greater vascular and anti-inflammatory activity than the metabolites formed and absorbed in the small intestine (30–33). In addition, the gut microbiome has an enormous capacity to regulate and modify host physiology, with growing evidence from germ-free mice of a role in energy metabolism and other cardiometabolic biomarkers (34). These gut microbes and their resulting metabolic products, like SCFAs and bile acids, can further influence risk factors for metabolic disease through their impact on energy metabolism or inflammation (35, 36), which together with anthocyanin-derived metabolites have been shown to affect physiological processes linked to metabolic disease, including fat partitioning, thermogenesis, and brown adipose tissue (37). After consumption of a highly plant-based diet, these microbiome-related activities have the potential both individually and synergistically to confer potential health benefits.

In agreement with our findings of an association between higher intake of foods rich in anthocyanins and higher abundance of Clostridiales and Ruminococcaceae, a human dietary intervention (*n*= 32) with 300 g fresh berries (containing mean ± SD 70.7 ± 52 mg anthocyanins) daily for 8 wk induced changes in the composition of the Lachnospiraceae and Ruminococcaceae families (38). Furthermore, grape seed extract and black raspberries have been shown to increase the abundances of Lachnospiraceae, Clostridiales, and Ruminococcacceae in animal feeding studies (39, 40). Lachnospiraceae and Ruminococcaceae are butyrate‐producers detected in >70% of individuals (41). Butyrate is known to protect from diet-induced obesity by enhancing intestinal barrier function, thereby inhibiting passage of proinflammatory molecules into the systemic circulation and preventing the occurrence of metabolic endotoxemia (42, 43). Butyrate has also been shown to have a direct effect on adipose tissue by promoting uncoupling protein-1 expression and adaptive thermogenesis in mice with depleted microbiota (37). Evidence from the current study, and others (40), therefore suggests that an increase in butyrate-producing bacteria may be a plausible mechanism underpinning the association between higher intake of foods rich in anthocyanins and lower adipose tissue. We do acknowledge, however, that the presence of butyrate-producing bacteria may not directly correlate with circulating or gut concentrations of butyrate owing to a number of factors, including carbohydrate availability and the relative abundance of different butyrate-producing bacteria.

We observed an association between higher intake of anthocyanin-rich foods and lower abundance of Clostridium XIVa. We also found increased abundances of Clostridium XIVa were associated with higher VAT. Taxonomy is defined in the current study using the Ribosomal Database Project database in which Clostridium XIVa is distinct from the Clostridium XIVa cluster as defined by Collins et al. (44) and does not encompass major butyrate-producing taxa such as *Roseburia* and *Coprococcus* that form separate genera (41, 45). Of interest, we found a significant association between higher intake of anthocyanin-rich foods and higher abundance of the butyrate-producing taxon *Roseburia*, although the finding did not reach statistical significance after adjusting for multiple testing. In agreement with our findings, *Clostridium coccoides* (Clostridium XIVa) was decreased after energy restriction and increased physical activity for 10 wk in overweight adolescents (46) and increased in obese compared with lean rodents (47). Another study also showed that vegetarian diets could decrease the abundance of Clostridium cluster XIVa species (48).

Although the associations between microbial diversity and intakes of anthocyanins and anthocyanin rich-foods were comparable, we did not find an association between estimated anthocyanin intake and abundances of specific taxa. Anthocyanin-rich foods included strawberries, red currants, blackberries, blueberries, and red wine, whereas anthocyanin intake comprised more diverse food sources. Mean ± SD anthocyanin intakes in this cohort were 39.0 ± 36.1 mg/d for men and 44.5 ± 46.0 mg/d for women. These intakes are comparable with those reported in EPIC-Germany (49). We found fruit to be a larger contributor (75.7%) and wine a smaller contributor (3.2%) to anthocyanin intake than previously reported for Central European countries (52.9% and 14.4%, respectively) (49). Anthocyanins from different plants have been shown to strongly affect gastrointestinal bacterial profiles (50) and phenolic–gut interactions will be influenced by food matrixes and interactions with other food components (51). Further research is needed to further understand the relative impact of anthocyanins per se on specific taxa.

Our finding that higher intakes of anthocyanins were independently associated with lower volumes of VAT and VAT:SAT ratio may be clinically relevant. We found that VAT was 493 cm^3^ lower in the highest than in the lowest consumers of anthocyanins, corresponding to 68.7 mg anthocyanins (equivalent to one-third of a cup of blueberries). In the Framingham Heart study an additional 500 cm^3^ VAT was associated with a 30% increase in risk of hypertension and a 76% increased risk of metabolic syndrome (52). We reported associations between higher anthocyanin intake and lower VAT but not SAT. Previous research suggests that anthocyanins significantly lower mesenteric adipose tissue, a component of VAT, but not de novo lipogenesis enzymes, which make a greater contribution to the intracellular pool of fatty acids in SAT than in VAT (53).

Strengths of the current study include the large sample of well-characterized participants, the direct measurement and quantification of adipose tissue using MRI (including the use of an unbiased postprocessing approach), and the assessment of all major flavonoid subclasses. We considered a range of important known confounders of variation in the gut microbiome including intakes of dietary fiber and fat. The following limitations merit consideration: this was a cross-sectional study so we were unable to infer causation from these findings and could not determine whether the interactions between anthocyanin intake and the gut microbiome were a factor in the development of VAT or secondary to the obesity itself. We did not measure circulating or gut concentrations of butyrate so we were not able to assess whether concentrations of butyrate differed according to anthocyanin intake. Although FFQs have been shown to accurately rank participants according to intakes of flavonoid-rich foods and our FFQ captured the main sources of flavonoids present in the habitual diet, it may not have captured all sources and measurement errors are inevitable (54). Furthermore, anthocyanin-rich foods are also an important source of other phytochemicals such as ellagitannins and resveratrol. Residual or unmeasured confounding is possible despite our detailed adjustment for a range of dietary and lifestyle confounder variables. Finally, owing to the different properties of microbial species within families, classes, or phyla, by grouping sequences we may have missed significant associations with both flavonoid intakes and volumes of adipose tissue.

In conclusion, we have shown, for the first time, that participants with the highest intakes of foods rich in anthocyanins had lower amounts of adipose tissue, greater microbial diversity, higher abundances of Clostridales and Ruminococcaceae, and a lower abundance of Clostridium XIVa than those with the lowest intakes. Furthermore, the microbial diversity and abundance of these taxa were associated with lower amounts of VAT. We estimated that ≤18.5% of the association between intake of anthocyanin-rich foods and VAT could be explained by the gut microbiome. Evidence from these novel data suggests that an increase in microbial diversity and abundance of specific taxa in the Clostridiales order may be a plausible mechanism underpinning the association between higher intake of anthocyanins and lower body fat.

## Supplementary Material

nqz299_Supplemental_File

## Notes

Supported in part by Biotechnology and Biological Sciences Research Council grant BB/J004545/1 (to AC), Deutsche Forschungsgemeinschaft (German Research Foundation) grant EXC 22167-390884018 (to WL) under Germany's Excellence Strategy, and German Federal Ministry of Education and Research grant 01GR0468. The PopGen 2.0 network is supported by German Federal Ministry of Education and Research grant 01EY1103 (to WL) and the Medical Faculty of the University of Kiel.

Supplemental Figures 1 and 2 are available from the “Supplementary data” link in the online posting of the article and from the same link in the online table of contents at https://academic.oup.com/ajcn/.

Data described in the article, code book, and analytic code will be made available upon request pending application and approval. The data set analyzed in the current study was obtained from the PopGen Biobank (Schleswig-Holstein, Germany) and can be accessed through a Material Data Access Form (https://portal.popgen.de/). Further information about this form and how to apply can be found at http://www.uksh.de/p2n/Information+for+researchers.html.

Abbreviations used: CRP, C-reactive protein; EER, estimated energy requirement; EPIC, European Prospective Investigation into Cancer and Nutrition; Q, quantile; SAT, subcutaneous abdominal adipose tissue; T, tertile; VAT, visceral abdominal adipose tissue.

## References

[1] Rothschild D, Weissbrod O, Barkan E, Kurilshikov A, Korem T, Zeevi D, Costea PI, Godneva A, Kalka IN, Bar N et al. Environment dominates over host genetics in shaping human gut microbiota. Nature. 2018;555:210–15. 29489753 10.1038/nature25973

[2] Zhernakova A, Kurilshikov A, Bonder MJ, Tigchelaar EF, Schirmer M, Vatanen T, Mujagic Z, Vila AV, Falony G, Vieira-Silva S et al. Population-based metagenomics analysis reveals markers for gut microbiome composition and diversity. Science. 2016;352:565–9. 27126040 10.1126/science.aad3369 PMC5240844

[3] Wang J, Thingholm LB, Skiecevičienė J, Rausch P, Kummen M, Hov JR, Degenhardt F, Heinsen FA, Rühlemann MC, Szymczak S et al. Genome-wide association analysis identifies variation in vitamin D receptor and other host factors influencing the gut microbiota. Nat Genet. 2016;48:1396–406. 27723756 10.1038/ng.3695 PMC5626933

[4] Cassidy A, Minihane AM The role of metabolism (and the microbiome) in defining the clinical efficacy of dietary flavonoids. Am J Clin Nutr. 2017;105:10–22. 27881391 10.3945/ajcn.116.136051 PMC5183723

[5] Manach C, Scalbert A, Morand C, Rémésy C, Jiménez L Polyphenols: food sources and bioavailability. Am J Clin Nutr. 2004;79:727–47. 15113710 10.1093/ajcn/79.5.727

[6] Queipo-Ortuño MI, Boto-Ordóñez M, Murri M, Gomez-Zumaquero JM, Clemente-Postigo M, Estruch R, Cardona Diaz F, Andrés-Lacueva C, Tinahones FJ Influence of red wine polyphenols and ethanol on the gut microbiota ecology and biochemical biomarkers. Am J Clin Nutr. 2012;95:1323–34. 22552027 10.3945/ajcn.111.027847

[7] Tzounis X, Rodriguez-Mateos A, Vulevic J, Gibson GR, Kwik-Uribe C, Spencer JP Prebiotic evaluation of cocoa-derived flavanols in healthy humans by using a randomized, controlled, double-blind, crossover intervention study. Am J Clin Nutr. 2011;93:62–72. 21068351 10.3945/ajcn.110.000075

[8] Anhê FF, Roy D, Pilon G, Dudonné S, Matamoros S, Varin TV, Garofalo C, Moine Q, Desjardins Y, Levy E et al. A polyphenol-rich cranberry extract protects from diet-induced obesity, insulin resistance and intestinal inflammation in association with increased *Akkermansia* spp. population in the gut microbiota of mice. Gut. 2015;64:872–83. 25080446 10.1136/gutjnl-2014-307142

[9] Roopchand DE, Carmody RN, Kuhn P, Moskal K, Rojas-Silva P, Turnbaugh PJ, Raskin I Dietary polyphenols promote growth of the gut bacterium *Akkermansia**muciniphila* and attenuate high-fat diet–induced metabolic syndrome. Diabetes. 2015;64:2847–58. 25845659 10.2337/db14-1916 PMC4512228

[10] Hughes LA, Arts IC, Ambergen T, Brants HA, Dagnelie PC, Goldbohm RA, van den Brandt PA, Weijenberg MP; Netherlands Cohort Study. Higher dietary flavone, flavonol, and catechin intakes are associated with less of an increase in BMI over time in women: a longitudinal analysis from the Netherlands Cohort Study. Am J Clin Nutr. 2008;88:1341–52. 18996871 10.3945/ajcn.2008.26058

[11] Bertoia ML, Rimm EB, Mukamal KJ, Hu FB, Willett WC, Cassidy A Dietary flavonoid intake and weight maintenance: three prospective cohorts of 124 086 US men and women followed for up to 24 years. BMJ. 2016;352:i17. 26823518 10.1136/bmj.i17 PMC4730111

[12] Jennings A, MacGregor A, Spector T, Cassidy A Higher dietary flavonoid intakes are associated with lower objectively measured body composition in women: evidence from discordant monozygotic twins. Am J Clin Nutr. 2017;105:626–34. 28100511 10.3945/ajcn.116.144394 PMC5320412

[13] Burke AC, Sutherland BG, Telford DE, Morrow MR, Sawyez CG, Edwards JY, Drangova M, Huff MW Intervention with citrus flavonoids reverses obesity and improves metabolic syndrome and atherosclerosis in obese *Ldl**r*^−^^/^^−^ mice. J Lipid Res. 2018;59:1714–28. 30008441 10.1194/jlr.M087387 PMC6121922

[14] Krawczak M, Nikolaus S, von Eberstein H, Croucher PJ, El Mokhtari NE, Schreiber S PopGen: population-based recruitment of patients and controls for the analysis of complex genotype-phenotype relationships. Community Genet. 2006;9:55–61. 16490960 10.1159/000090694

[15] Müller HP, Raudies F, Unrath A, Neumann H, Ludolph AC, Kassubek J Quantification of human body fat tissue percentage by MRI. NMR Biomed. 2011;24:17–24. 20672389 10.1002/nbm.1549

[16] Lindauer E, Dupuis L, Müller HP, Neumann H, Ludolph AC, Kassubek J Adipose tissue distribution predicts survival in amyotrophic lateral sclerosis. PLoS One. 2013;8:e67783. 23826340 10.1371/journal.pone.0067783 PMC3694869

[17] Kaess BM, Pedley A, Massaro JM, Murabito J, Hoffmann U, Fox CS The ratio of visceral to subcutaneous fat, a metric of body fat distribution, is a unique correlate of cardiometabolic risk. Diabetologia. 2012;55:2622–30. 22898763 10.1007/s00125-012-2639-5 PMC3636065

[18] Kroke A, Klipstein-Grobusch K, Voss S, Möseneder J, Thielecke F, Noack R, Boeing H Validation of a self-administered food-frequency questionnaire administered in the European Prospective Investigation into Cancer and Nutrition (EPIC) study: comparison of energy, protein, and macronutrient intakes estimated with the doubly labeled water, urinary nitrogen, and repeated 24-h dietary recall methods. Am J Clin Nutr. 1999;70:439–47. 10500011 10.1093/ajcn/70.4.439

[19] USDA. USDA database for the flavonoid content of selected foods: release 2.1. Washington (DC): USDA; 2007.

[20] USDA. USDA database for the proanthocyanidin content of selected foods. Washington (DC): USDA; 2004.

[21] Dehne LI, Klemm C, Henseler G, Hermann-Kunz E The German food code and nutrient data base (BLS II.2). Eur J Epidemiol. 1999;15:355–9. 10414376 10.1023/a:1007534427681

[22] Kozich JJ, Westcott SL, Baxter NT, Highlander SK, Schloss PD Development of a dual-index sequencing strategy and curation pipeline for analyzing amplicon sequence data on the MiSeq Illumina sequencing platform. Appl Environ Microbiol. 2013;79:5112–20. 23793624 10.1128/AEM.01043-13 PMC3753973

[23] Magoc T, Salzberg SL FLASH: fast length adjustment of short reads to improve genome assemblies. Bioinformatics. 2011;27:2957–63. 21903629 10.1093/bioinformatics/btr507 PMC3198573

[24] Edgar RC, Haas BJ, Clemente JC, Quince C, Knight R UCHIME improves sensitivity and speed of chimera detection. Bioinformatics. 2011;27:2194–200. 21700674 10.1093/bioinformatics/btr381 PMC3150044

[25] Wang Q, Garrity GM, Tiedje JM, Cole JR Naive Bayesian classifier for rapid assignment of rRNA sequences into the new bacterial taxonomy. Appl Environ Microbiol. 2007;73:5261–7. 17586664 10.1128/AEM.00062-07 PMC1950982

[26] Edgar RC UPARSE: highly accurate OTU sequences from microbial amplicon reads. Nat Methods. 2013;10:996–8. 23955772 10.1038/nmeth.2604

[27] Ainsworth BE, Haskell WL, Herrmann SD, Meckes N, Bassett DR Jr, Tudor-Locke C, Greer JL, Vezina J, Whitt-Glover MC, Leon AS 2011 Compendium of Physical Activities: a second update of codes and MET values. Med Sci Sports Exerc. 2011;43:1575–81. 21681120 10.1249/MSS.0b013e31821ece12

[28] Institute of Medicine of the National Academies. Dietary Reference Intakes for energy, carbohydrate, fiber, fat, fatty acids, cholesterol, protein and amino acids. Washington (DC): National Academies Press; 2005.

[29] Rodriguez-Mateos A, Istas G, Boschek L, Feliciano RP, Mills CE, Boby C, Gomez-Alonso S, Milenkovic D, Heiss C Circulating anthocyanin metabolites mediate vascular benefits of blueberries: insights from randomized controlled trials, metabolomics, and nutrigenomics. J Gerontol A Biol Sci Med Sci. 2019;74(7):967–76. 30772905 10.1093/gerona/glz047

[30] di Gesso JL, Kerr JS, Zhang Q, Raheem S, Yalamanchili SK, O'Hagan D, Kay CD, O'Connell MA Flavonoid metabolites reduce tumor necrosis factor-α secretion to a greater extent than their precursor compounds in human THP-1 monocytes. Mol Nutr Food Res. 2015;59:1143–54. 25801720 10.1002/mnfr.201400799 PMC4973837

[31] Amin HP, Czank C, Raheem S, Zhang Q, Botting NP, Cassidy A, Kay CD Anthocyanins and their physiologically relevant metabolites alter the expression of IL-6 and VCAM-1 in CD40L and oxidized LDL challenged vascular endothelial cells. Mol Nutr Food Res. 2015;59:1095–106. 25787755 10.1002/mnfr.201400803 PMC4950056

[32] de Ferrars RM, Czank C, Zhang Q, Botting NP, Kroon PA, Cassidy A, Kay CD The pharmacokinetics of anthocyanins and their metabolites in humans. Br J Pharmacol. 2014;171:3268–82. 24602005 10.1111/bph.12676 PMC4080980

[33] Czank C, Cassidy A, Zhang Q, Morrison DJ, Preston T, Kroon PA, Botting NP, Kay CD Human metabolism and elimination of the anthocyanin, cyanidin-3-glucoside: a ^13^C-tracer study. Am J Clin Nutr. 2013;97:995–1003. 23604435 10.3945/ajcn.112.049247

[34] Everard A, Geurts L, Caesar R, Van Hul M, Matamoros S, Duparc T, Denis RG, Cochez P, Pierard F, Castel J et al. Intestinal epithelial MyD88 is a sensor switching host metabolism towards obesity according to nutritional status. Nat Commun. 2014;5:5648. 25476696 10.1038/ncomms6648 PMC4268705

[35] Kasubuchi M, Hasegawa S, Hiramatsu T, Ichimura A, Kimura I Dietary gut microbial metabolites, short-chain fatty acids, and host metabolic regulation. Nutrients. 2015;7:2839–49. 25875123 10.3390/nu7042839 PMC4425176

[36] Fiorucci S, Distrutti E Bile acid-activated receptors, intestinal microbiota, and the treatment of metabolic disorders. Trends Mol Med. 2015;21:702–14. 26481828 10.1016/j.molmed.2015.09.001

[37] Li B, Li L, Li M, Lam SM, Wang G, Wu Y, Zhang H, Niu C, Zhang X, Liu X et al. Microbiota depletion impairs thermogenesis of brown adipose tissue and browning of white adipose tissue. Cell Rep. 2019;26:2720–37..e5. 30840893 10.1016/j.celrep.2019.02.015

[38] Puupponen-Pimiä R, Seppänen-Laakso T, Kankainen M, Maukonen J, Törrönen R, Kolehmainen M, Leppänen T, Moilanen E, Nohynek L, Aura AM et al. Effects of ellagitannin-rich berries on blood lipids, gut microbiota, and urolithin production in human subjects with symptoms of metabolic syndrome. Mol Nutr Food Res. 2013;57:2258–63. 23934737 10.1002/mnfr.201300280

[39] Choy YY, Quifer-Rada P, Holstege DM, Frese SA, Calvert CC, Mills DA, Lamuela-Raventos RM, Waterhouse AL Phenolic metabolites and substantial microbiome changes in pig feces by ingesting grape seed proanthocyanidins. Food Funct. 2014;5:2298–308. 25066634 10.1039/c4fo00325j PMC4744461

[40] Pan P, Lam V, Salzman N, Huang Y-W, Yu J, Zhang J, Wang L-S Black raspberries and their anthocyanin and fiber fractions alter the composition and diversity of gut microbiota in F-344 rats. Nutr Cancer. 2017;69:943–51. 28718724 10.1080/01635581.2017.1340491 PMC6139254

[41] Vital M, Karch A, Pieper DH Colonic butyrate-producing communities in humans: an overview using omics data. mSystems. 2017;2:e00130–17. 29238752 10.1128/mSystems.00130-17 PMC5715108

[42] Brahe LK, Astrup A, Larsen LH Is butyrate the link between diet, intestinal microbiota and obesity-related metabolic diseases?. Obes Rev. 2013;14:950–9. 23947604 10.1111/obr.12068

[43] Peng L, Li ZR, Green RS, Holzman IR, Lin J Butyrate enhances the intestinal barrier by facilitating tight junction assembly via activation of AMP-activated protein kinase in Caco-2 cell monolayers. J Nutr. 2009;139:1619–25. 19625695 10.3945/jn.109.104638 PMC2728689

[44] Collins MD, Lawson PA, Willems A, Cordoba JJ, Fernandez-Garayzabal J, Garcia P, Cai J, Hippe H, Farrow JA The phylogeny of the genus *Clostridium*: proposal of five new genera and eleven new species combinations. Int J Syst Bacteriol. 1994;44:812–26. 7981107 10.1099/00207713-44-4-812

[45] Lozupone C, Faust K, Raes J, Faith JJ, Frank DN, Zaneveld J, Gordon JI, Knight R Identifying genomic and metabolic features that can underlie early successional and opportunistic lifestyles of human gut symbionts. Genome Res. 2012;22:1974–84. 22665442 10.1101/gr.138198.112 PMC3460192

[46] Santacruz A, Marcos A, Wärnberg J, Martí A, Martin-Matillas M, Campoy C, Moreno LA, Veiga O, Redondo-Figuero C, Garagorri JM et al. Interplay between weight loss and gut microbiota composition in overweight adolescents. Obesity (Silver Spring). 2009;17:1906–15. 19390523 10.1038/oby.2009.112

[47] Jiao N, Baker SS, Nugent CA, Tsompana M, Cai L, Wang Y, Buck MJ, Genco RJ, Baker RD, Zhu R et al. Gut microbiome may contribute to insulin resistance and systemic inflammation in obese rodents: a meta-analysis. Physiol Genomics. 2018;50:244–54. 29373083 10.1152/physiolgenomics.00114.2017

[48] Matijašić BB, Obermajer T, Lipoglavšek L, Grabnar I, Avguštin G, Rogelj I Association of dietary type with fecal microbiota in vegetarians and omnivores in Slovenia. Eur J Nutr. 2014;53:1051–64. 24173964 10.1007/s00394-013-0607-6

[49] Zamora-Ros R, Knaze V, Luján-Barroso L, Slimani N, Romieu I, Touillaud M, Kaaks R, Teucher B, Mattiello A, Grioni S et al. Estimation of the intake of anthocyanidins and their food sources in the European Prospective Investigation into Cancer and Nutrition (EPIC) study. Br J Nutr. 2011;106:1090–9. 21481290 10.1017/S0007114511001437

[50] Overall J, Bonney SA, Wilson M, Beermann A III, Grace MH, Esposito D, Lila MA, Komarnytsky S Metabolic effects of berries with structurally diverse anthocyanins. Int J Mol Sci. 2017;18:422. 10.3390/ijms18020422 PMC5343956 28212306

[51] Sengul H, Surek E, Nilufer-Erdil D Investigating the effects of food matrix and food components on bioaccessibility of pomegranate (*Punica granatum*) phenolics and anthocyanins using an *in-vitro* gastrointestinal digestion model. Food Res Int. 2014;62:1069–79.

[52] Lee JJ, Pedley A, Hoffmann U, Massaro JM, Fox CS Association of changes in abdominal fat quantity and quality with incident cardiovascular disease risk factors. J Am Coll Cardiol. 2016;68:1509–21. 27687192 10.1016/j.jacc.2016.06.067 PMC5599249

[53] Hoek-van den Hil EF, van Schothorst EM, van der Stelt I, Swarts HJ, van Vliet M, Amolo T, Vervoort JJ, Venema D, Hollman PC, Rietjens IM et al. Direct comparison of metabolic health effects of the flavonoids quercetin, hesperetin, epicatechin, apigenin and anthocyanins in high-fat-diet-fed mice. Genes Nutr. 2015;10:469. 26022682 10.1007/s12263-015-0469-z PMC4447677

[54] Carlsen MH, Karlsen A, Lillegaard IT, Gran JM, Drevon CA, Blomhoff R, Andersen LF Relative validity of fruit and vegetable intake estimated from an FFQ, using carotenoid and flavonoid biomarkers and the method of triads. Br J Nutr. 2011;105:1530–8. 21272408 10.1017/S0007114510005246

