# Supplementary material for: The role of the gut microbiome in the association between habitual anthocyanin intake and visceral abdominal fat in population-level analysis
Source: Am J Clin Nutr. 2019 Dec 11;111(2):340–50. doi: 10.1093/ajcn/nqz299 (PMC6997102; doi:10.1093/ajcn/nqz299)
Supplement: nqz299_Supplemental_File [file nqz299_supplemental_file.docx]

**Supplementary Figure 1:** **Flow chart of the PopGen study population**

Study participants from the PopGen control cohort (recruited between 2005 and 2007)

**n= 1316**

Lost to Follow-up (n= 364)

First follow-up examination (2010-2012)

**n= 929**

Attended for MRI examination

**n= 656**

Incomplete MRI data or containing artefacts (n= 30)

Missing FFQ data (n= 1)

Missing microbiome data (n= 7)

Included in analyses

**n= 618**

**Supplementary Figure 2: Relative abundance of gut microbiota genera in n=618 PopGen participants**

Bars represent the unadjusted means (error bars standard deviation) of taxa, grouped according to genus (family), that made up the core measureable dataset.
